# Supplementary material for: The interactome of intact mitochondria by cross-linking mass spectrometry provides evidence for coexisting respiratory supercomplexes
Source: Mol Cell Proteomics. 2017 Dec 8;17(2):216–32. doi: 10.1074/mcp.RA117.000470 (PMC5795388; doi:10.1074/mcp.RA117.000470)
Supplement: Supplemental Data [file supp_RA117.000470_133922_0_supp_23978_fzffwf.zip › spectra_annotation/mito_DR_spectra_annotation/450-1-12-1-20-1.pdf]

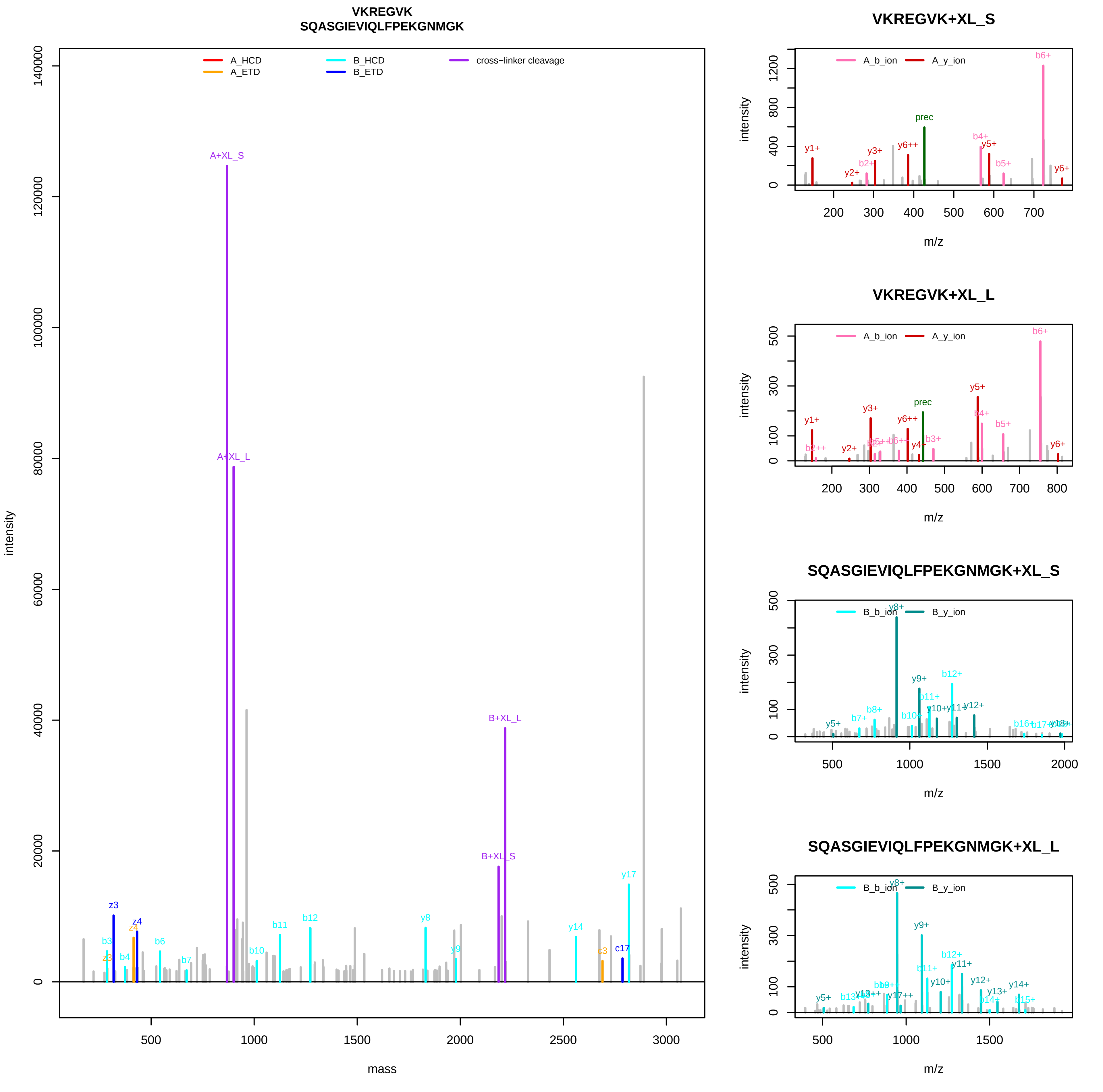
VKREGVK
SQASGIEVIQLFPEKGNMGK
A_HCD
A_ETD
B_HCD
B_ETD
cross−linker cleavage
A+XL_S
A+XL_L
B+XL_L
B+XL_S
z3
z4
b3
z3
b4
b6
b7
b10
b11
b12
y8
y9
y14
c3
c17
y17
intensity
mass
0
20000
40000
60000
80000
100000
120000
140000
500
1000
1500
2000
2500
3000
VKREGVK+XL_S
A_b_ion
A_y_ion
y1+
y2+
b2+
y3+
y6++
prec
b4+
y5+
b5+
b6+
y6+
intensity
m/z
VKREGVK+XL_L
A_b_ion
A_y_ion
y1+
b2++
y2+
y3+
y6++
y4+
b3+
prec
y5+
b4+
b5+
b6+
y6+
intensity
m/z
SQASGIEVIQLFPEKGNMGK+XL_S
B_b_ion
B_y_ion
y8+
y5+
b7+
b8+
b10+
y9+
b11+
b12+
y10+
y11+
y12+
b16+
b17+
y18+
intensity
m/z
SQASGIEVIQLFPEKGNMGK+XL_L
B_b_ion
B_y_ion
y8+
y9+
y5+
b13+
y13++
b10+
y17++
b11+
y10+
b12+
y11+
y12+
b14+
y13+
y14+
b15+
intensity
m/z
